# Intrathecal Injection of Spironolactone Attenuates Radicular Pain by Inhibition of Spinal Microglia Activation in a Rat Model

**DOI:** 10.1371/journal.pone.0039897

**Published:** 2012-06-29

**Authors:** Yu-e Sun, Liangyu Peng, Xiaofeng Sun, Jinhua Bo, Dong Yang, Yaguo Zheng, Chenglong Liu, Beibei Zhu, Zhengliang Ma, Xiaoping Gu

**Affiliations:** Department of Anaesthesiology, Affiliated Drum-Tower Hospital of Medical College of Nanjing University, Nanjing, Jiangsu province, China; Hokkaido University, Japan

## Abstract

**Background:**

Microglia might play an important role in nociceptive processing and hyperalgesia by neuroinflammatory process. Mineralocorticoid receptor (MR) expressed on microglia might play a central role in the modulation of microglia activity. However the roles of microglia and MR in radicular pain were not well understood. This study sought to investigate whether selective MR antagonist spironolactone develop antinociceptive effects on radicular pain by inhibition neuroinflammation induced by spinal microglia activation.

**Results:**

Radicular pain was produced by chronic compression of the dorsal root ganglia with SURGIFLO™. The expression of microglia, interleukin beta (IL-1β), interleukin 6 (IL-6), tumor necrosis factor alpha (TNF-α), NR1 subunit of the NMDA receptor (t-NR1), and NR1 subunit phosphorylated at Ser896 (p-NR1) were also markedly up-regulated. Intrathecal injection of spironolactone significantly attenuated pain behaviors as well as the expression of microglia, IL-1β, TNF-α, t-NR1, and p-NR1, whereas the production of IL-6 wasn’t affected.

**Conclusion:**

These results suggest that intrathecal delivery spironolactone has therapeutic effects on radicular pain in rats. Decreasing the activation of glial cells, the production of proinflammatory cytokines and down-regulating the expression and phosphorylation of NMDA receptors in the spinal dorsal horn and dorsal root ganglia are the main mechanisms contributing to its beneficial effects.

## Introduction

Chronic low back pain is a significant clinical problem for which current treatments are inadequate [1]. This is due in large part to the fact that the mechanisms underlying neuropathic allodynia and hyperalgesia are insufficiently understood. Increasing evidence strongly implicates a role for spinal glia and proinflammatory cytokines, such as IL-1β, TNF-α and IL-6, in the genesis or maintenance of persistent pain via central mechanism [2]–[5]. Microglial cells are the innate immune cells of the central nervous system, they respond quickly to injury and produce a wide variety of proinflammatory molecules, neurotrophic factors, and neurotransmitters [6]. Mounting evidence indicates that microglia actively communicate with neurons and are important contributors in the development of neuropathic pain [7]–[8]. Spinal expressions of IL-1β, TNF-α, and IL-6 are elevated in association with pain facilitation [5] and intrathecal administration of cytokines also produce allodynia, hyperalgesia and changes in spinal cord neuronal responses to nociceptive stimuli in the rat [9]. Revealed by both western blot experiments and patch-clamp recordings, activated microglia and proinflammatory cytokines increased the expression and phosphorylation of NMDA receptor, enhanced NMDA-induced currents and contributed to neuropathic pain and proinflammatory pain [3], [4], [10], [11], [28]. Our previous study also found a remarkable inflammatory process in the spinal dorsal horn and dorsal root ganglia (DRG) after chronic compression of dorsal root ganglia in rats [12]. In addition, intrathecal therapy with anti-inflammatory prednisolone acetate inhibited pain behaviors and the upregulation of neuronal nitric oxide synthase (nNOS) and NMDA receptor in the spinal dorsal horn [13].

NMDA receptors are heteromeric complexes incorporating different subunits chosen within a repertoire of three subtypes: NR1, NR2 and NR3. Of particular importance is the high permeability to calcium ions, which confers on NMDA receptors a central role in synaptic plasticity [14]. Numerous studies have reported that the expression and activation of NMDA receptors in the spinal dorsal horn play a crucial role in the development and maintenance of acute and persistent pain [15]–[18]. Our previous studies also found that there were marked increases in the expression of NMDA receptor in the superficial dorsal horn and DRG after chronic compression of the dorsal root ganglia [12], [13], [19]. Intrathecal injection of NMDA receptor antagonist ifenprodil attenuated thermal hyperalgesia and mechanical allodynia induced by chronic compression of the dorsal root ganglia or bone cancer [19], [20].

Spironolactone is a competitive aldosterone receptor antagonist used for over 40 years to treat diseases associated with primary or secondary hyperaldosteronism [21]. Growing preclinical and clinical evidences suggest that treatment of microglia with GCs decreases the ability of these cells to proliferate [22], to produce proinflammatory cytokines [23]. Evidence suggests that spironolactone inhibits production of several proinflammatory cytokines, including TNF-α and IL-1β via mineralocorticoid receptor or non-mineralocorticoid receptor mechanisms and show positive effects in patients with immunoinflammatory diseases [24]–[27]. An in vivo study showed that corticosterone and aldosterone at concentrations lower than 1 nM enhanced the activities of microglial cells which could be reversed by spironolactone [28]. Frieler et al. found that MR knockout resulted in significant reduction in activated microglia, the production of proinflammatory cytokines and infarct volume after middle cerebral artery occlusion [29]. Recent studies shown that spironolactone exerts therapeutic effects on neuropathic pain and capsaicin-induced chemogenic pain [30], [31]. In light of these reports, it is possible that spironolactone may inhibit the activation of spinal glial cells and decrease the production of proinflammatory cytokines. Subsequently, the expression and function of NMDA receptors in the spinal dorsal horn were affected. Our previous studies also found that intrathecal delivery of spironolactone has therapeutic effects on radicular pain [32].

**Figure 1:**
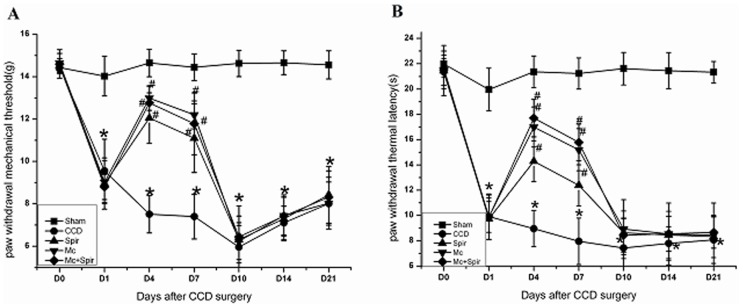
Intrathecal injection of spironolactone (Spir) improves pain behaviors after CCD surgery (n = 6/each). D0, 1, 4, 7, 10, 14, 17 and 21 indicate days of CCD. All data points represent mean±SD. Compared with sham group, chronic compression of the dorsal root ganglion (CCD) significantly decreased paw withdraw mechanical threshold (PWMT) (A) and thermal latency (PWTL) (B), ^*^ *P*<0.01. Both mechanical allodynia (A) and thermal hyperalgesia (B) were diminished in CCD rats treated with spironolactone (3 µg), minocycline (0.11 mg) and spironolactone (3 µg) with half-hour pre-application minocycline (0.11 mg) twice a day from Days 2 to 4 subsequent to CCD surgery, ^#^ *P<*0.01.

In our present study, we examined the hypothesis that spironolactone inhibits the activation of glial cells and the production of proinflammatory cytokines, the expression and phosphorylation of NMDA receptor in spinal dorsal horn and DRG, and exerts beneficial effects on pain behaviors in a rat model of radicular pain induced by chronic compression of lumbar dorsal root ganglia with SURGIFLO™.

## Results

### Intrathecal Injection of Spironolactone Improves Pain Behaviors after CCD Surgery

Consist with our previous study, both mechanical allodynia (Figure 1A) (*P*<0.001) and thermal hyperalgesia (Figure 1B) (*P*<0.001) were observed on the ipsilateral side following CCD surgery and lasted up to at least 21 days. Spironolactone (3 µg), minocycline (0.11 mg), spironolactone (3 µg) with half-hour pre-application minocycline (0.11 mg) were administered intrathecally twice a day from Days 2 to 4 subsequent to CCD surgery significantly reduced paw withdrawal mechanical threshold on Day 4 [(12.05±1.19) g, (12.99±0.58) g, (12.77±0.78) g ] VS (7.51±0.88) g, *P*<0.01, and Day 7 [(11.10±1.62) g, (12.20±0.66) g, (11.78±1.46) g ] VS (7.41±1.06) g, *P*<0.01 (Figure 1A) as well as paw withdrawal thermal latency on Day 4 [(14.29±1.61) s, (16.99±1.58) s, (17.71±1.48) s ] VS (8.96±1.43) s, *P*<0.01, and Day 7 [(12.39±1.62) s, (15.20±1.66) s, (15.78±1.46) s] VS (7.97±1.84) s, *P*<0.01 (Figure 1B) in comparison to vehicle-treated CCD rats. The selected spironolactone dose has been proven to have analgesic effect in CCD rats [36]. However, no significant difference was found on the contralateral side and between the minocycline (0.11 mg) and spironolactone (3 µg) with half-hour pre-application minocycline (0.11 mg) (*P*>0.05, data not shown). Based on these obtained results we come to the conclusion that spironolactone plays a pivotal role in relieving pain hypersensitivity via inactivation of microglia in our animal model.

### Intrathecal Injection of Spironolactone Inhibits the Expression of Microglia in the Spinal Dorsal Horn

**Figure 2:**
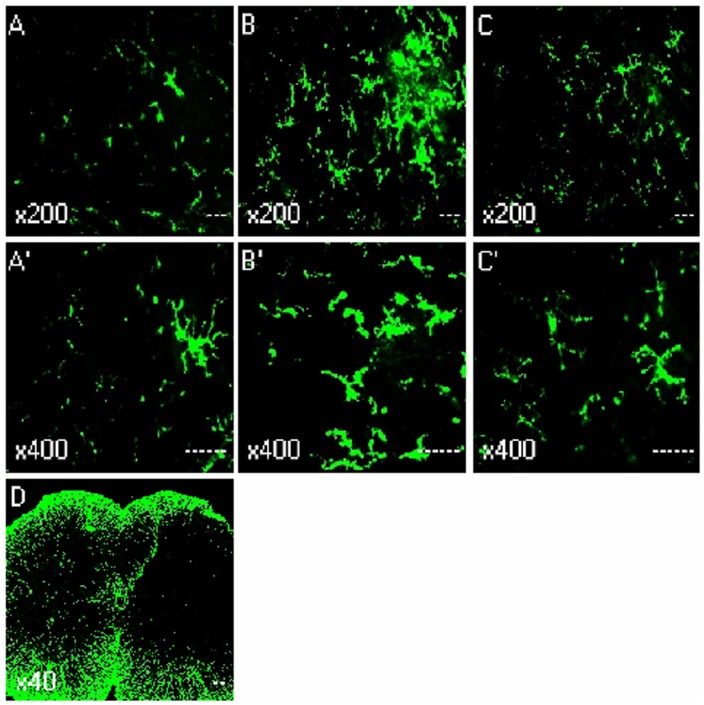
Immunohistochemistry showed the effects of intrathecal injection of spironolactone on activation of microglia in the spinal dorsal horn (n = 3/each). Quantification of microglia activation after CCD surgery. Confocal images of sham (A and A’), Day 4 after CCD surgery (B, B’ and D), Day 4 after injection of spironolactone (C and C’), showing the increase in the microglia marker OX-42 in L4 spinal cord sections, the activated microglia cells typically exhibited hypertrophy with thicker processes, larger and densely stained cell bodies after CCD surgery. Injection of spironolactone inhibited microglia activation.

**Figure 3:**
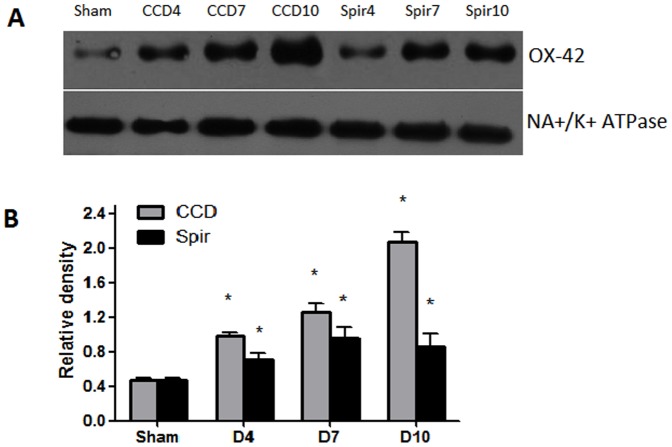
Weston blot and statistical analysis showed the effects of intrathecal injection of spironolactone on activation of microglia in the spinal dorsal horn (n = 3/each). The expression and activation of microglia (OX-42) were significantly up-regulated after CCD surgery compared with Sham group, ^*^ *p*<0.01 (A and B). Intrathecal delivery of spironolactone (3 µg) twice a day from Days 2 to 4 subsequent to CCD surgery significantly inhibited the expression of microglia on Day 4 and Day 7 after CCD surgery, ^#^ *p*<0.05, ^##^ *p*<0.01 (A and B).

Figure 2 and 3 illustrated the changes in the expression of microglia in spinal dorsal horn after CCD surgery and the time-course effects of spironolactone. To test whether the activation of microglia in the spinal dorsal horn, we performed immunostaining and western blot with anti-OX-42 antibody. Compared with the contralateral, the microglia marker OX-42 was significant increase in spinal cord sections, which were ipsilateral to the CCD surgery (Fig. 2D). Compared with the sham rats (Fig. 2A and 2A’), the activated microglia cells typically exhibited hypertrophy with thicker processes, larger and densely stained cell bodies after CCD surgery (Fig. 2B and 2B’). Injection of spironolactone inhibited microglia activation (Fig. 2C and 2C’). The upregulation of OX-42 was quantified by Western blot. The tissues from the spinal cord sections were punched out, and total proteins were isolated. As shown in Figure 3, consistent with pain behaviors, remarkable up-regulation of microglia was observed after CCD surgery in the spinal dorsal horn (Figure 3A–B). Intrathecal delivery of spironolactone significantly suppressed the expression of microglia on Day 4 [(0.67±0.10) VS (1.18±0.12), *P = *0.016] and Day 7 [(1.02±0.17) VS (1.56±0.13), *P = *0.026].

### Spironolactone Inhibits the Production of Proinflammatory Cytokines in the Spinal Dorsal Horn and DRG

**Figure 4:**
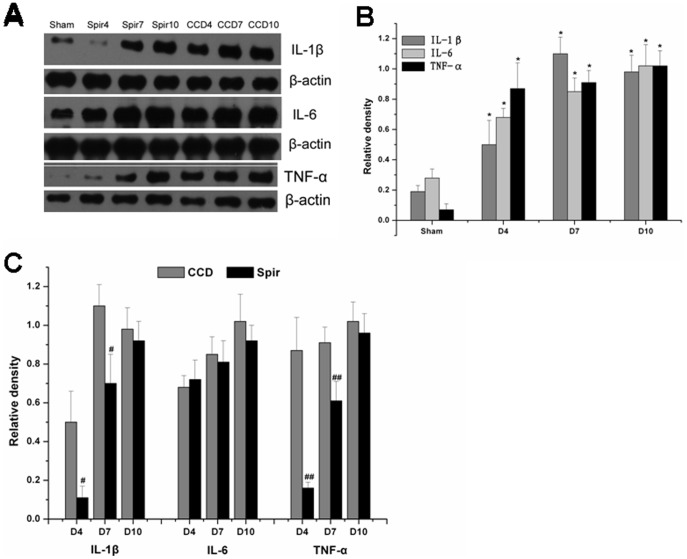
Weston blot and statistical analysis showed the effects of intrathecal injection of spironolactone on production of proinflammatory cytokines in the spinal dorsal horn (n = 3/each). After CCD surgery, the expression of IL-1β, IL-6 and TNF-α were significantly up-regulated compared with Sham group, ^*^ *P*<0.01 (A and B). Intrathecal injection of spironolactone (3 µg) twice a day from Days 2 to 4 subsequent to CCD surgery significantly reduced the production of IL-1β and TNF-α (A and C) compared with vehicle treated CCD rats, ^#^ *P*<0.05, ^##^ *P*<0.01. Spironolactone has no effects on the production of IL-6 compared with vehicle treated CCD rats, *P*>0.05 (A and C).

**Figure 5:**
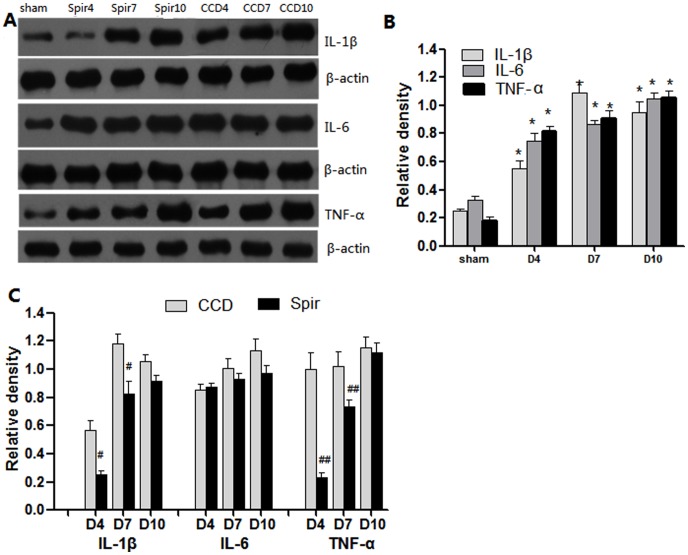
Weston blot and statistical analysis showed the effects of intrathecal injection of spironolactone on production of proinflammatory cytokines in the DRG (n = 3/each). After CCD surgery, the expression of IL-1β, IL-6 and TNF-α were significantly up-regulated compared with Sham group, ^*^ *P*<0.01 (A and B). Intrathecal injection of spironolactone (3 µg) twice a day from Days 2 to 4 subsequent to CCD surgery significantly reduced the production of IL-1β and TNF-α (A and C) compared with vehicle treated CCD rats, ^#^ *P*<0.05, ^##^ *P*<0.01. Spironolactone has no effects on the production of IL-6 compared with vehicle treated CCD rats, *P*>0.05 (A and C).

Figure 4 and 5 illustrated the changes in the expression of proinflammatory cytokines in spinal dorsal horn and DRG after CCD surgery and the time-course effects of spironolactone. Consistent with pain behaviors, remarkable up-regulation of IL-1β, IL-6 and TNF-α were observed after CCD surgery in the spinal dorsal horn and DRG (Figure 4A–B and 5A–B). Intrathecal delivery of spironolactone significantly suppressed the production of IL-1β and TNF-α on Day 4 [IL-1 β: (0.11±0.06) VS (0.50±0.16), *P = *0.019; TNF-α: (0.16±0.03) VS (0.87±0.17), *P = *0.002], [IL-1β: (0.22±0.09) VS (0.58±0.11), *P* = 0.026; TNF-α: (0.23±0.04) VS (1.07±0.15), *P* = 0.001] and Day 7 [IL-1 β: (0.70±0.15) VS (1.10±0.11), *P* = 0.020; TNF-α: (0.61±0.10) VS (0.91±0.08), *P* = 0.014], [IL-1β: (0.81±0.12) VS (1.20±0.13), *P* = 0.019; TNF-α: (0.73±0.08) VS (1.11±0.12), *P* = 0.015], while no notable effect was seen on the expression of IL-6 (*P>*0.05) (Figure 4A, 4C and 5A, 5C ).

### Spironolactone Decreases the Expression and Phosphorylation of NMDA Receptor

**Figure 6:**
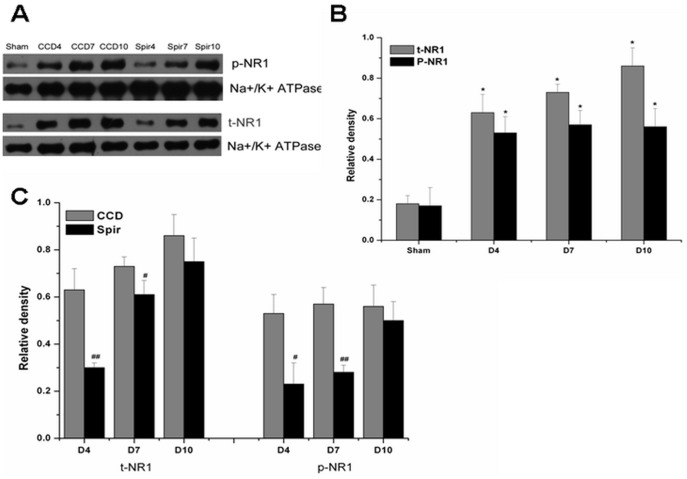
Weston blot and statistical analysis showed the effects of intrathecal injection of spironolactone on the expression and phosphorylation of NMDAR in the spinal dorsal horn (n = 3/each). The expression and phosphorylation of NMDA receptor NR1 subunit (t-NR1 and p-NR1) were significantly up-regulated after CCD surgery compared with Sham group, ^*^ *p*<0.01 (A and B). Intrathecal delivery of spironolactone (3 µg) twice a day from Days 2 to 4 subsequent to CCD surgery significantly inhibited the expression of t-NR1 and p-NR1 on Day 4 and Day 7 after CCD surgery, ^#^ *p*<0.05, ^##^ *p*<0.01 (A and C).

**Figure 7:**
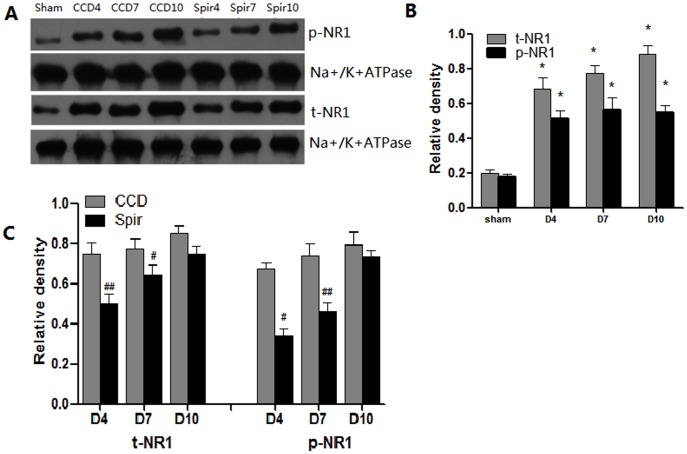
Weston blot and statistical analysis showed the effects of intrathecal injection of spironolactone on the expression and phosphorylation of NMDAR in the DRG (n = 3/each). The expression and phosphorylation of NMDA receptor NR1 subunit (t-NR1 and p-NR1) were significantly up-regulated after CCD surgery compared with Sham group, ^*^ *p*<0.01 (A and B). Intrathecal delivery of spironolactone (3 µg) twice a day from Days 2 to 4 subsequent to CCD surgery significantly inhibited the expression of t-NR1 and p-NR1 on Day 4 and Day 7 after CCD surgery, ^#^ *p*<0.05, ^##^p<0.01 (A and C).

It has been reported that proinflammatory enhanced excitatory synaptic transmission was one of the main mechanisms involved in central sensitization [3], [4]. Thus we further examined the expression and phosphorylation of NMDA receptor in spinal dorsal horn and DRG. Congruous with pain behaviors and the expression of proinflammatory cytokines, there were marked elevation of total NR1 (t-NR1) and phosphor-NR1 at Ser896 (p-NR1) in spinal dorsal horn and DRG after CCD surgery. When treated with spironolactone, as shown in figure 6 and 7, the expression of t-NR1 and p-NR1 were significantly decreased on Day 4 [t-NR1: (0.30±0.02) VS (0.63±0.09), *P* = 0.004; p-NR1: (0.23±0.09) VS (0.53±0.08), *P* = 0.012], [t-NR1: (0.46±0.03) VS (0.70±0.07), *P* = 0.005; p-NR1: (0.32±0.12) VS (0.67±0.13), *P* = 0.004] and Day 7 [t-NR1: (0.61±0.06) VS (0.73±0.04), *P* = 0.046; p-NR1: (0.28±0.03) VS (0.57±0.07), *P* = 0.002] [t-NR1: (0.60±0.04) VS (0.80±0.09), *P* = 0.021; p-NR1: (0.48±0.03) VS (0.77±0.02), *P* = 0.012].

## Discussion

The primary findings of this study are that chronic compression of the dorsal root ganglia with SURGIFLO™ induced long-lasting thermal hyperalgesia and mechanical allodynia, led to robust increase in IL-β, IL-6 and TNF- α, activated the microglia and elevated the expression and phosphorylation of NMDA receptors within the spinal dorsal horn and DRG ipsilateral to CCD. Intrathecal treatment with spironolactone twice daily on day 2–4 after CCD surgery markedly improved pain behaviors, had no effect on pain behavior under inhibition of glial cells by pre-application of minocycline, down-regulated the expression of IL-1βand TNF-α and inhibited the activation of microglia. The expression and phosphorylation of NMDA receptor also significantly decreased in the spinal dorsal horn and DRG. Nevertheless, spironolactone exhibited no influence on the expression of IL-6. Our results suggest that microglia are a source of multiple cytokines, including IL-1β, IL-6 and TNF-α, which can contribute to different features of pathological pain. It also confirmed that microglial cells is not the only source of IL-6. At the same time, IL-6 can also be derived from other cells, such as astrocytes. Taken together, we conclude that spironolactone inhibits the activation of microglia, down-regulates the production of proinflammatory cytokines as well as inhibits the expression and activation of NMDA receptors in the spinal dorsal horn and DRG are the main mechanisms contributing to improving pain behaviors.

Consistent with our previous study, radicular pain hyperexcitability can be alleviated via intrathecal injection of spironolactone following chronic compression of the dorsal root ganglia [32], whereas Wang et al. [37], [38] reported that spironolactone given intrathecally had no effects on neuropathic pain after peripheral nerve injury in rats. This apparent discrepancy probably arises from the different study models used in the three papers. Studies suggest that peripheral injury activates glia and leads to the induction of cytokines and other chemical mediators in glial cells. Cytokines are then released and act back on neurons to facilitate central sensitization, activated glial cells are a source of cytokines [4]. The present study demonstrates interactions between microglia and neurons through proinflammatory cytokines and NMDAR coupling in central hyperexcitability and inflammatory hyperalgesia. The expressions of several proinflammatory cytokines, as revealed by western blot experiments in our present study, are considerably increased following chronic compression of the dorsal root ganglia. Our previous experiment also revealed a notable up regulation of Iκβ-α in the early stage of radicular pain [12]. The expression of Iκβ-α parallels with NF-κβ activity, which has been proposed to be a reliable marker for cytokine-responsive cells within the central nervous system [39], [40]. While no overt tissue inflammation and edema occurred at the spinal level [37], [41], together with the expression of NF-κβ in the superficial dorsal horn, on the contrary, shown marked decrease after peripheral nerve injury [42]. Moreover, epidural or intrathecal administration with anti-inflammatory glucocorticoids exerted beneficial effects on pain behaviors and significantly decreased the expression of NMDA receptor in the superficial dorsal horn and DRG after chronic compression of the dorsal root ganglia [12], [13], whereas intrathecal treatment with dexamethasone exacerbated neuropathic pain behaviors after peripheral nerve injury [37], [38]. Correlated with their study, the expression of NMDA receptor did not change after intrathecal injection of spironolactone. These opposing results further confirm that the anti-inflammatory potency of spironolactone in the superficial dorsal horn and DRG is implicated in the mechanism for which beneficial effects on radicular pain behaviors are involved.

A large body of animal and clinical studies also reported that spironolactone has potent anti-inflammatory effects in multiple organs and systems via mineralocorticoid receptor or non-mineralocorticoid receptor mechanisms. For example, first, in principal cells of the cortical collecting duct, spironolactone attenuated aldosterone-induced inflammatory cytokines production such as IL-1β and IL-6 via mineralocorticoid receptor-dependent pathway [43], second, in human mononuclear cells, spironolactone inhibited the activation of NF-κβ and the expression of several NF-κβ-targeted genes via non-mineralocorticoid receptor mechanisms [44], [45], third, in rheumatoid arthritis patients, treatment with spironolactone improves both endothelial dysfunction and inflammatory disease activity [24], [27]. In our present study, we also found a robust anti-inflammatory effect of spironolactone. However, whether these effects are mineralocorticoid receptor-dependent or non-mineralocorticoid receptor-relevant need further exploration. Though an in vivo study showed that spironolactone can reduce the activation of glial cells through mineralocorticoid receptor dependent pathway [28], the expression of mineralocorticoid receptors in glial cells in spinal cord and DRG after CCD surgery remains to be investigated.

To test our hypothesis that spinal dorsal horn and DRG glial activation, inflammatory cytokine release as well as the expression and phosphorylation of NMDA receptor affect or facilitate neuronal plasticity through interactions with mineralocorticoid receptor and play a critical role in the development of inflammatory hyperalgesia, we studied the effects of mineralocorticoid receptor antagonist spironolactone. Our results shown that intrathecal injection of spironolactone significantly down-regulated the expression and phosyphorylation of NMDA receptors in the spinal dorsal horn and DRG, while Wang and his colleagues found that spironolactone had no effects on the expression of NMDA receptors in the spinal cord. The anti-inflammatory effect of spironolactone may underlie the main mechanisms contributing to its inhibitory action on the expression and phosphorylation of NMDA receptors in the superficial dorsal horn. Our data showed that, as revealed by western blot experiments, spironolactone significantly down-regulated p-NR1 and t-NR1 on Day 4 and 7 after CCD surgery. Notably, the time-course of NMDA receptor expression and phosphorylation generally parallel that of proinflammatory cytokines, microglia and pain behaviors changes after intrathecal injection of spironolactone in rats. Numerous in vitro and in vivo evidences also suggest the existence of a functional interaction among microglia, proinflammatory cytokines and NMDA receptors. For example, (1) IL-1β enhanced NMDA receptor NR1 subunit phosphorylation in the inflammatory pain models [4] and cancer pain models [10] while intrathecal injection IL-1 receptor antagonist can alleviate inflammatory hyperalgesia via preventing phosphorylation of NMDA receptor NR1 subunit in rats [11], (2) through activation of tyrosine kinases and subsequent NR2A/B subunit phosphorylation, IL-1β can significantly increased NMDA receptor function in hippocampal neurons [46], (3) recently, TNF-α and IL-1β were shown to increase AMPA and NMDA-induced currents in lamina II superficial dorsal horn neurons [3], (4) via sphingomyelin phosphodiesterase 3 pathway, TNF-α can enhance the expression and phosphorylation of NMDA receptors [47]. Though previous studies shown that the activation of MRs produce striking facilitation of long-term potentiation [48], a recent study proved that these effects are mediated by AMPA receptor but not NMDA receptors [49]. However, under present experiment condition, we can not deny the direct modulation effects of spironolactone on the expression and phosphorylation of NMDA receptors in the spinal cord of radicular pain rat.

Jaqqi et al. found that systemic administration of spironolactone significantly attenuated chronic constriction injury-induced pain related behaviors and the production of TNF-α in the sciatic nerve [31]. Our previous studies shown that remarked inflammatory processes and significant NMDA upregulation also occurred in the dorsal root ganglion [12]. Intrathecal spironolactone injection may potentially influence dorsal root ganglion and regions beyond the targeted spinal segments. However, many previous researches suggest that intrathecal injection of drugs in a low volume is a method of regional drug delivery, only effects on homologous spinal segments [38], [50], [51]. In our present experiments, IL-1beta, TNF-alpha, t-NR1 and phosphorylated NR1 are also expressed in DRG, suggesting a potential role of them in pain processing in the DRG. Intrathecal administration of spironolactone inhibited the up-regulation of IL-1beta, TNF-alpha, t-NR1 and phosphorylated NR1 in the DRG as well as the spinal dorsal horn. So the primary site of spironolactone action should be within the spinal cord and DRG in the present experiments.

The present findings may have important implication. Following its peripheral anti-inflammatory effects was explored we further found that spironolactone can significantly inhibits the activation of microglia, decrease the production of proinflammatory cytokines as well as the expression and phosphorylation of NMDA receptors at the spinal level. Advancing from previous studies, the model emphasizes activation of glia by tissue injury, concomitant cytokine release, and post-translational regulation of NMDAR through IL-1R signaling. In addition, many clinical studies had proved that both intravenous and oral spironolactone is reasonably safe and economically attractive. Spironolactone may be an ideal anti-inflammatory drug used to treat low back pain in the near future.

### Conclusion

In summary, the present study shown that IL-1beta, TNF-alpha, t-NR1 and phosphorylated NR1 were involved in pathogenesis of neuropathic pain not only in the spinal cord but also in the DRG, and intrathecal delivery of spironolactone has therapeutic effects on radicular pain induced by chronic compression of the dorsal root ganglion with SURGIFLO™. Spironolactone inhibits the activation of microglia, down-regulates the production of IL-1β and TNF-α as well as decreases the expression and phosyphorylation of NMDA receptors are the main mechanisms contributing to its beneficial effects. However, whether spironolactone inhibits the activation of microglia and the production of proinflammatory cytokines is mineralocorticoid receptor dependent or not still unknown and whether spironolactone participates in the regulation other type of glutamate receptors more than NMDA receptors also need further studies to investigate.

## Methods

### Animal Models of Radicular Pain

Adult male Sprague Dawley rats weighing 275–325 g (provided by the Laboratory Animal Center of Drum Tower Hospital) were used. The animal room was artificially lighted from 7:00 A.M. until 7:00 P.M. Rats were housed in individual cages with free access to water and food pellets. Room temperature was maintained at 24°C. The experimental protocol was approved by our Institutional Animal Care and Use Committee. Aseptic technique was used in all surgical procedures. For CCD operation, rats had their right L5 DRG/exiting nerve root compressed by a hemostatic matrix (SURGIFLO™) according to the method we described previously [12]. Briefly, the surgical procedure was performed aseptically under pentobarbital anesthesia (50 mg/kg intraperitoneally). Paraspinal muscles were separated from the mammillary process and the transverse process to expose the right L5 intervertebral foramen. A stainless 22-G steel needle, with a blunt angle to avoid tissue penetration, was gently inserted approximately 4 mm into the L5 intervertebral foramen. The needle was inserted at a 30–40°angle toward the dorsal middle line and 10–15°below the vertebral horizontal line. Care was taken to minimize contact with the existing nerve root/DRG by monitoring twitches of ipsilateral hind paw muscles. When small twitches were noted, the needle was either slightly withdrawn or redirected. SURGIFLO™ (60 µl) was slowly injected (within 1–2 min) into the right L5 intervertebral foramen, which was often accompanied by one or two mild twitches of ipsilateral hind paw muscles. After the injection, the muscle and skin layers were closed with 6.0 nylon sutures. Sham operation was performed following the same surgical procedure but without the SURGIFLO™ injection. The animals were treated with an intraperitoneal injection of cefuroxime in the prophylactic dose of 20 mg/kg, 2 h preoperatively and once a day for the next 2 days to prevent infection.

### Intrathecal Catheterization and Drug Delivery

An intrathecal PE10 catheter was implanted in a rat under the same surgical conditions to the level of the lumbar enlargement (∼8.5 cm from the incision site for this rat age group) according to the method described previously [33]. Those rats exhibiting postoperative neurological deficits (e.g., paralysis) or poor grooming were excluded from the experiments. Spironolactone and minocycline were also purchased from Sigma (St. Louis, MO) and dissolved in 10% ethanol solution. Spironolactone and minocycline were injected intrathecally in a 10 µl volume followed by a 10 µl saline flush.

### Immunohistochemistry

Rats were deeply anesthetized with pentobarbital sodium (100 mg/kg, i.p.) and perfused transcardially with 4% paraformaldehyde in 0.1 M phosphate buffer at pH 7.4. The lumbar spinal cord and the DRG segments were removed, postfixed, and transferred to 30% sucrose for frozen sections. After washes in PBS, the sections were blocked for 60 min at room temperature with 10% (v/v) normal fetal bovine serum (Vector Laboratories, Peterborough, UK) in PBS. The primary Ab (rabbit polyclonal anti- OX-42, 1∶200, chemicon, CA) was applied, and samples were then incubated for 48 h at 4°C. After washing, the secondary Ab (Alexa Fluor 488, 1∶500, abcam, CA) was applied, and samples were further incubated at 4°C overnight. Sections were mounted on glass slides, air-dried and coverslipped using Aquamount (Fisher Scientific, Ottawa, Canada). Images were taken at 40×, 200× and 400× magnification using the Leica TCS SP2 multiphoton confocal microscope (Leica Microsystems, Wetzlar, Germany).

### Western Blot

Rats were killed rapidly by decapitation after anesthesia, Fresh tissue samples from bilateral L5 DRG and spinal cord dorsal horn were removed after laminectomy. Lumbar spinal and DRG segments were divided into the ipsilateral and contralateral side as well as the dorsal and ventral part and homogenized in an SDS sample buffer containing a mixture of proteinase inhibitors (Sigma, USA). Protein samples were separated on SDS-PAGE gel (OX-42,IL-1 β, IL-6 and TNF-α: 12% gradient gel, t-NR1 and p-NR1: 6% gradient gel) and transferred to polyvinylidene difluoride filters (Millipore, USA). The filters were blocked with 5% milk and then immunoblotted using antibodies against OX-42 (Abcam, USA, 1∶500 dilution), IL-1 β (Bioworld, USA, 1∶400 dilution), IL-6 (Santa cruz, USA, 1∶400 dilution), TNF-α (R&D Systems, USA, 1∶400 dilution), NR1 (abcam, UK, 1∶500 dilution), and NR1 (phospho Ser896) (abcam, UK, 1∶500 dilution). The membrane was washed with TBS and incubated with goat polyclonal secondary antibody to rabbit IgG (abcam, UK, 1∶5000 dilution). The blots were visualized in ECL solution (DuPont-NEN, USA) for 1 min and exposed to hyperfilms (Amersham Biosciences) for 1–10 min. The density of specific bands was measured with a computer-assisted imaging analysis system and normalized against corresponding loading control bands. β-actin (abcam, UK, 1∶1000 dilution) was used as a loading control for IL-1β, IL-6 and TNF-α, while Na+/K+ ATPase (abcam, UK, 1∶500 dilution) for NR1 and NR1 (phospho Ser896).

### Behavioral Tests

Animals were habituated to the test environment daily (a 60 min session) for 3 days before baseline testing. The testing procedure for thermal hyperalgesia was performed according to a previously published method [34]. Temperature was set to achieve baseline latency at 12 s and a cutoff of 25 s. Mechanical allodynia was examined by applying a von Frey filament to the plantar surface of each hindpaw [35]. The cut off force was 15 g.

### Statistical Analysis

All data are expressed as mean ±SD (standard deviation). Data from behavioural tests were analyzed by using repeated ANOVA measurements across testing time points to detect overall differences among treatment groups. Data from western blot experiments were analyzed by using One-way ANOVA to determine differences among all experiment groups. In both cases, when significant main effects were observed, the LSD post hoc tests were performed to determine sources of differences. Differences were considered to be statistically significant at the level of α = 0.05.
